# Accelerating learning for pro-poor health markets

**DOI:** 10.1186/1744-8603-10-54

**Published:** 2014-06-24

**Authors:** Sara Bennett, Gina Lagomarsino, Jeffrey Knezovich, Henry Lucas

**Affiliations:** 1 Johns Hopkins School of Public Health, Baltimore, MD, USA; 2 Results for Development, Washington, DC, USA; 3 Institute of Development Studies, University of Sussex, Brighton, UK

**Keywords:** Health care market, Learning, Developing countries

## Abstract

**Background:**

Given the rapid evolution of health markets, learning is key to promoting the identification and uptake of health market policies and practices that better serve the needs of the poor. However there are significant challenges to learning about health markets. We discuss the different forms that learning takes, from the development of codified scientific knowledge, through to experience-based learning, all in relationship to health markets.

**Discussion:**

Notable challenges to learning in health markets include the difficulty of acquiring data from private health care providers, designing evaluations that capture the complex dynamics present within health markets and developing communities of practice that encompass the diverse actors present within health markets, and building trust and mutual understanding across these groups.

The paper proposes experimentation with country-specific market data platforms that can integrate relevant evidence from different data sources, and simultaneously exploring strategies to secure better information on private providers and health markets. Possible approaches to adapting evaluation designs so that they are better able to take account of different and changing contexts as well as producing real time findings are discussed. Finally capturing informal knowledge about health markets is key. Communities of practice that bridge different health market actors can help to share such experience-based knowledge and in so doing, may help to formalize it. More geographically-focused communities of practice are needed, and such communities may be supported by innovation brokers and/or be built around member-based organizations.

**Summary:**

Strategic investments in and support to learning about health markets can address some of the challenges experienced to-date, and accelerate learning that supports health markets that serve the poor.

## Background

A health market consists of the set of interactions that occur between multiple health actors – such as health care providers, payors, regulators and service users – and the rules and regulations that govern their actions and interactions. Providers, payors and regulators may be either public or private entities operating either on a for-profit or on a not-for-profit basis. Health markets in low and middle income countries (LMICs) are currently experiencing extremely rapid change due to technological and organizational innovation, as well as political transformation [1]. For example, social franchising schemes that provide specific health services such as family planning services or TB care are expanding rapidly in countries [2,3], and sometimes even crossing national borders. As governments roll out social health insurance schemes, private for-profit providers may be attracted into new health care markets by the increased ability of the population to afford health care [4]. The revolution in information and communications technology has provided opportunities to create networks across health care providers, sometimes linking informal health care providers with private telemedicine centers [5]. For many years the predominant focus of health systems research has been the public health care sector, accordingly evidence concerning what happens in the private sector is relatively weak. Now, change in health markets is occurring so rapidly that typically there is insufficient time (not to mention funding) to formally evaluate the effectiveness of new interventions.

This paper focuses on strategies for strengthening learning about health markets in the future. While from a business perspective a focus on the bottom line may be sufficient, from a public health perspective, more information is needed about how changes in the market affect people’s access to quality health services. Such information would help governments and investors to promote good practices, and particularly those that improve access to care for the poor. There have been several recent efforts to promote the development of learning communities around health markets^a^, however this paper argues that many gaps remain, and that further effort and resources need to be invested in learning about health markets.

**Figure 1:**
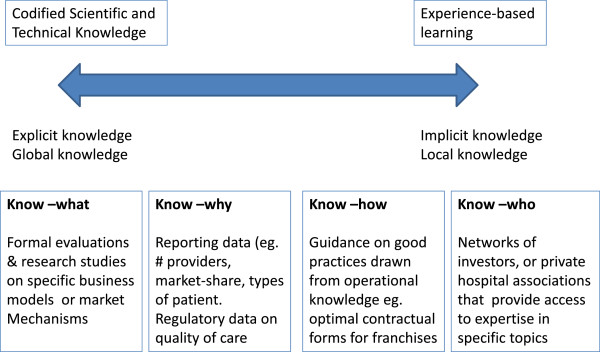
Different forms of knowledge and their relevance to health markets.

Learning is a broad term, and we use it here advisedly. Learning can encompass the acquisition of knowledge or skills through experience, practice, study or taught courses and may take the form of individual learning, organizational learning or even learning across systems [6-10]. In recent years the health sector has seen a surge of interest in promoting the application of formal research evidence to decision making [11,12], but management specialists have focused more on the contribution of implicit and tacit evidence to learning and decision making [13]. Figure 1 unpacks the multiple forms of evidence and learning that are needed to promote strong and efficient health markets, from formal studies that seek to evaluate the effects of interventions, through more descriptive, empirical information that describes what is going on in health markets and hence why certain actions or interventions might be required, to more implicit or experience-based forms of learning.

Given the dearth of previous literature concerning learning about health markets, we have purposefully maintained a broad focus in our paper. We aim to provide an overview of the landscape of health market learning, thus providing a foundation and stimulation for others to dig deeper into particular aspects. The paper proceeds by describing barriers to more effective learning about health markets and then identifies and elaborates two separate, but related strategies to address learning challenges. The first focuses on approaches to generating evidence and knowledge about health markets. The second considers mechanisms for supporting learning across different types of actors in health markets. Finally we conclude with practical recommendations about how to move the agenda forward.

## Main text

### Barriers to learning in health markets

We consider challenges to (i) knowledge building, which concerns how knowledge regarding health markets is generated, and is particularly focused on more formal knowledge (e.g. evaluations, research) and (ii) knowledge sharing and evidence use, which concerns the interactions between different actors and the application of knowledge to decisions, and is focused more on the exchange of implicit and best practice knowledge.

### Challenges to knowledge building

It can be very challenging to acquire data from private, particularly for-profit, health care providers. Few such providers are included in government reporting systems, and their information is often incomplete and unreliable [14]. Even private providers operating fully within government regulations are often reluctant to share sensitive data (financial performance, services provided, etc.) because of concerns that it may, *inter alia*, be disclosed to competitors, or to officials able to inhibit activities or seek bribes. Moreover, in many countries the poor obtain care mainly from unlicensed or moonlighting public sector providers [15]. Both groups are unlikely to provide information that might be passed to government agencies.

While relevant data on private providers may be obtained from household surveys, those data are typically limited in scope and often unreliable [16]. Respondents cannot assess technical quality of care, and often cannot reliably report services provided, fees paid, provider qualifications or even if providers were public or private. Exit or mystery patient surveys [17,18] can provide additional insights, but demand-side surveys alone cannot deliver detailed understanding of the diverse range of private providers.

Even with access to reliable information, evaluating health market initiatives is challenging. They typically involve many interconnected parts and players within a larger system and have potential consequences well beyond the intended health outcomes: the science of designing such evaluations is poorly understood and rarely practiced [19]. For example, Shah et al. [20] present a rigorous and systematic approach to evaluating family planning franchises, but their framework does not address the wider implications of such interventions, including how franchised services affect the quality or price of similar services on offer from non-franchised providers. While other conceptual models allow consideration of the broader ramifications of market interventions [21], these have rarely been translated into solid empirical studies^b^.

Another key challenge concerns contextual factors: the internal management context of an organization (including leadership, financial systems etc.); the health market context (including market concentration, regulatory environment, access to capital), and the broader socio-economic context (including GNP per capita, extent of formal sector employment) may all influence the effectiveness of a market intervention. Many evaluations take inadequate account of such factors and thus it is difficult to assess the transferability of findings to other contexts.

Further, impact evaluations typically take years to implement, and technologies and business models often evolve more quickly than the evidence base. Many evaluations are of pilot exercises and often ignore the fact that the challenges of implementing the same intervention at scale may be different in nature and considerably more complex. Moreover, pilots may be feasible for evaluation of new business models but are often of limited value in understanding initiatives designed to influence the market – such as regulatory, tax and incentive policies – which are, arguably, the interventions where knowledge needs are greatest.

Similarly, evaluations typically have time horizons of one to three years and do not explore how interventions may give rise to unexpected side-effects over the longer run as stakeholders adjust to a new environment, shift the long term balance of market power, or shape market institutions in ways that may have long lasting effects. Given the significance of path dependence in health systems [22], these issues cannot be ignored. In particular, any specific health market constellation will create interest groups likely to develop entrenched positions and political power over time, and fight later attempts at reform or regulation [23].

A final set of issues concerns the institutional arrangements for documenting market experiences: frequently no provision is made to fund for independent evaluation, and the implementers of initiatives are made responsible for evaluating their own performance and documenting effects. This raises serious concerns about bias and the trustworthiness of data.

### Challenges to knowledge sharing and evidence use

Health markets in LMICs are dominated by multiple, small-scale, poorly coordinated actors. In a complex system such as a health market, no single actor will have a complete view of the change processes occurring; so, there is a need to bring together different types of actors, including government, private providers, regulators, payors and market analysts, to develop shared learning. However there is a widespread belief that mutual distrust and skepticism about motivations are prevalent between public and private sector actors [24]. Without overcoming this mutual distrust, joint learning is unlikely to be successful.

Relatedly, different actors in health markets may hold very different paradigms about the nature of the knowledge and evidence required for decision-making: for example, private sector firms may place greater value on evidence of the impact they have on their customers or patients, rather than population-level outcomes. They may also look to financial indicators or market share as an indicator of success, whereas public health experts may seek impact evaluations based on established health indicators.

Finally practical questions persist about how best to develop organizational and institutional structures that promote the exchange and use of evidence regarding health markets. Recently, international actors have invested in the development of communities of practice that aim to share information across countries and across different types of actors, but these often face serious challenges in terms of language barriers, time pressures on participants, and maintaining up-to-date information. While new technologies might help mitigate some of these challenges to learning, there needs to be clearer and more systematic thinking about quite how to address such practical issues.

### Addressing challenges to knowledge building

#### Answering descriptive questions better

Country governments often lack the most basic descriptive data concerning health markets outside of the public sector: where are private providers located and what kinds of services and financing do they provide? To the extent that data do exist they typically only cover formal private sector providers (see Table 1).

**Table 1:** Sources of data on private health care providers

**Data source**	**Nature of data**	**Issues**
Household surveys (eg. Demographic and Health Surveys)	Provides household level data on utilization of private providers	Reliance on consumer recall for data on price, quality of services is problematic.
Private providers registered with MOH	Regulatory databases	Informal providers typically not included.
		Only basic data on formal providers collected.
		While many LMICs have regulatory frameworks for HMIS, enforcement is often partial leading to incomplete or out-of-date information.
Facility surveys (eg. Service Provision Assessment of DHS)	Survey of health facilities covering aspects such availability of services, staffing and resources, and actual services provided	Can include private providers but does not do so routinely.
		No regular roster of facility surveys established despite previous discussions among global actors about the importance of such information.
Demographic surveillance sites (eg. Matlab, Bangladesh; Kintampo, Ghana)	Frequent, regular collection of household health data from surveillance sites, including care seeking behavior	INDEPTH (the society of LMIC surveillance sites) is still working on developing better linkages between such data and facility surveys.
		While some surveillance data have explored private sector utilization, there remains considerable scope to expand further.
Data collected by payors such as insurance schemes	Typically encompasses data on patient characteristics, diagnosis, services provided, and price of care received	Partial coverage of health insurance schemes in low and middle income countries means that currently such databases typically cover only a small proportion of the population. Further data collected is designed to meet the needs of payors, rather than informing broader policy decisions.
Financial flows (eg. PETS, NHA)	PETS track the flow of government finance through the health care system. NHA includes components that look at private sector financing and expenditure in the private sector	NHA is relatively well developed with respect to the private sector. PETS typically only cover private sector actors, when public funds flow to them.
Routine Health Management Information systems	Routine data typically including health services data	Private providers typically not included, with the exception of notifiable diseases. Extent of enforcement of regulations on notifiable diseases is unclear.
Financial data	Data on company revenues, capital, profitability. Companies and formal private providers typically report to government for tax reasons	Infrequently used in research or linked with other data sources.

The Health Metrics Network framework for health information architecture in low- and middle-income countries [25] emphasizes the need for routine data collection from private health care providers. However, private for-profit providers are rarely incorporated in national information systems, and there has been little serious discussion of the feasibility of integrating private sector reporting. Strategies to improve market data include incentives for private providers to register with or provide health services data to government, or stronger consequences for failing to do so, or ensuring that a sample of private practitioners is included within routine health facility surveys. While global actors have discussed such strategies, to-date there has been little effort to support their implementation. An alternative approach that might be of interest, is that taken by the Center for Studying Health Systems Change [26]. The Center has conducted a series of longitudinal studies of health markets in 12 communities across the United States. Center research staff visit the communities every two-three years and employ primarily interviews with a range of different market stakeholders to describe and analyze changes in financing, organization and delivery of health care within these geographically defined markets.

In high-income countries that have large private sectors, public or private payors typically collect routine health information from both public and private health care providers. Because payment is contingent on reporting, provider incentives are aligned. The growing commitment to universal health coverage, often linked to the development of health insurance and social protection schemes in lower-income countries, provides a potential window of opportunity to strengthen market-oriented health management information systems to deliver the kind of data needed for public health policy.

### Answering effectiveness questions better

Recent years have seen a spate of systematic reviews related to health markets, a number of which are summarized in Table 2. In most cases review authors found a good number of studies that could be included in the review, however the quality of the primary studies included was heavily criticized. Most of the reviews address relatively self-contained interventions in the market that have been planned and implemented by the government or an NGO, such as contracting out of services by government, social franchising or social marketing initiatives. Strictly for-profit private sector initiatives appear less likely to be evaluated, partly because of the type of evidence that is valued in the commercial sector, but also because traditional evaluation approaches are ill-suited to private sector initiatives that are continuously evolving as entrepreneurs adapt strategies to changing market conditions. Further, relatively few of the reviews focus on market governance mechanisms (such as regulation).

**Table 2:** Key findings regarding the nature of evidence from recent systematic reviews

**Authors**	**Title**	**# studies included**	**Conclusions regarding nature of evidence available**
Patouillard et al. [27]	Can working with the private for-profit sector improve utilization of quality health services by the poor? A systematic review of the literature	52	The authors highlight that only a handful of studies assess the impact of private-sector involvement on usage and quality of health care for the poor. While many studies show increased access to health services for the poor, due to the quality of existing studies it is not possible to prove that private-sector involvement in health care is beneficial to the poor. The authors also recommend a focus on robust evaluation designs in future research, because current data are insufficient and of poor quality.
Berendes et al. [28]	Quality of private and public ambulatory health care in low and middle income countries: Systematic review of comparative studies	80	The authors stress the need for more research using standardised outcome measures, and assessing strategies and interventions, to improve private ambulatory health-care services.
Montagu et al. [29]	Private versus public strategies for health service provision for improving health outcomes in resource-limited settings	21	Overall the quality of the evidence was rated as either low or very low and the authors conclude there is a need for further evidence comparing health outcomes of public-sector versus private-sector health care.
Kiwanuka et al. [30]	Dual practice regulatory mechanisms in the health sector: A systematic review of approaches and implementation	31	Majority of studies identified were policy analyses, country case studies, cross-sectional surveys, or economic models. No impact evaluations were identified, and no studies assessed the impact of regulatory mechanisms on dual practice.
Liu et al. [31]	The effectiveness of contracting-out primary health care services in developing countries: A review of the evidence	16	The authors highlight the need for more research on the possible unanticipated consequences of contracting-out interventions. To-date very few evaluations have addressed these.
Koehlmoos et al. [32]	Social franchising evaluations: A scoping review	3 systematic reviews, 9 primary studies	The authors conclude that there is a lack of rigorous evaluations of the effectiveness of social franchising, and that future research should address issues related to implementation, such as adherence and sustainability.
Evans et al. [33]	Systematic review of public health branding	3 experimental studies 5 quasi-experimental 25 observational	The authors conclude that there are problems in the existing literature with reference to the standardization of reporting, terminology and measurement. They express the need for more rigorous research designs such as randomised controlled trials and longitudinal designs to determine the effectiveness of public health branding interventions on health behaviour.

Source: extracted from 3ie database of systematic reviews

With regard to evaluations of interventions in health markets, there is both a need to strengthen traditional impact evaluations and also to experiment with alternative types of evaluation. Traditional impact evaluations face numerous challenges, such as being commissioned too late or with insufficient funding [34]. Initiatives such as 3ie are beginning to tackle this, but there remain missed opportunities for learning as new private sector initiatives are often not identified in advance, and are started without concomitant investments in evaluation.

Further research is also needed in order to understand how context affects market interventions. Strengthened efforts to map contextual factors would help in terms of assessing the transferability of findings. The notion of developing a limited number of market archetypes deserves further exploration: for example, some health markets are heavily dominated by social health insurance schemes, others rely on public provision combined with out-of-pocket payment for health care, still others focus largely on private informal sector providers. If it were possible to develop a set of market archetypes, then policy and decision-makers would be able to situate their own health market among these archetypes and accordingly better understand the implications of research findings from elsewhere for their particular setting. This idea has been suggested before in the context of health systems [35], but has not been properly explored in the context of health markets.

Interventions in markets frequently encounter unexpected effects beyond their immediate targets [21]. Evaluation designs need to do a better job at measuring anticipated effects, but also searching for unanticipated ones, for example: have regulatory interventions shifted care seeking to the unregulated private sector? Have successful franchising networks, through competitive pressures, managed to increase the quality and lower the price of providers outside of the franchise? We need creative ways to capture rapid evolution in the nature of interventions, and the forces that have shaped this evolution, such as actor power, information exchange and market competition. Unfortunately there is little prior research concerning the spread of innovations in complex systems that can inform such studies [36].

Given the dynamic and adaptive nature of health markets (described above), evaluations need to track interventions over multiple time points rather than employing simple before and after studies. Demographic surveillance sites, with their frequent rounds of household data collection offer particular promise in this respect, especially if they could be linked with facility surveys. Establishing nationally representative, routine facility surveys that incorporate private providers would also enhance our ability to understand and evaluate the effects of health market interventions at scale. Further, given the dynamism of many health markets, there are frequently multiple different market-related interventions that may be occurring in overlapping areas of the country. Conducting controlled studies in such environments may be close to impossible, and as others have argued there is a need to examine how a national information platform could support evaluations in such contexts [37].

### Answering applied policy and practice questions better

Even with the findings of robust evaluations in hand, it is frequently difficult to address the kind of questions that policy-makers and practitioners are interested in, that often concern implementation processes, for example: how should we go about implementing a new regulatory regime? What challenges are we likely to face in contracting out specialist services? How do we set up quality assurance mechanisms in a franchise agreement? Answers to such questions need to draw upon the best research evidence available, both in terms of what has worked and why, and also the implicit, informal knowledge possessed by practitioners who have struggled with similar questions in their own contexts.

A major question concerns how to systematically collate implicit and informal knowledge. Much useful information on health markets is currently generated through market research and other non-academic, unpublished industrial analysis on a proprietary basis^c^. In commercial marketplaces, business consultants may be repositories of such information, having worked across multiple organizations, and assessed the effects of different business practices. Although staff of multilateral and bilateral development agencies and their contractors may possess some of this tacit knowledge, they are typically not in a position to acquire the kind of skills and expertise of market researchers, dedicated to a particular geographical market. Collaborations with market researchers may help to consolidate informal information about health markets.

While analysts often discuss separately the kind of processes required for formal research versus “learning by doing” (see Figure 1) [38], in practice these two different approaches to learning can complement each other and the interaction between formal and informal knowledge can drive the crystallization of implicit ideas in a process sometimes referred to as the SECI model [13]^d^. Historically the health sector has not been particularly good at facilitating communication between those focused on formal scientific evidence and those possessing tacit knowledge, but for the reasons described above, this seems to be a particularly important interface for health markets, and one that is discussed in more detail below in the section on facilitating learning.

### Addressing challenges to knowledge sharing

#### Communities of practice

Where solid, scientific knowledge about health markets exists it is perhaps best conveyed through structured, didactic training courses. Such courses are currently available as part of graduate training programs at universities across the world, and through short course training, such as that conducted by the World Bank Institute. However, where knowledge is evolving rapidly, and primarily exists in the form of tacit knowledge, training courses are unlikely to be very effective and establishing strong communities of practice (“know who”) may be more useful than transferring codified, explicit knowledge (“know what”). Creating connections between different actors interested in health markets may be key to stimulating innovation and the diffusion of promising new interventions. Developing communities of practice is particularly important for health markets where there is frequently distrust and a lack of communication across actors in different sectors and relevant actors reside in multiple different organizations (such as funding agencies, regulators, provider organizations). Lack of trust not only inhibits the development of communities of practice but can also interfere with learning as actors may be reluctant to test ideas or voice thoughts that are not yet fully developed [39].

Accordingly effective learning processes for health markets need to (i) combine formal, explicit knowledge with informal tacit knowledge and (ii) build communities of practice [4] that break down barriers that separate different types of health market actors.

New approaches to organizing learning events are emerging and these need to be encouraged and evaluated. For example, instead of short workshops or meetings, learning events may occur over a period of months, with online exchange building to a focal, face-to-face event, and then subsequent follow-up online, or perhaps an extended series of engagements in which the same group of actors meet on a regular basis [40]. Such an approach allows time for relationships to be built and for trust to develop. Collaborative learning, an approach already widely employed in formal educational settings, is increasingly being applied outside of formal educational settings to dispersed networks of people. For example a group of implementers operating in different locations but working on similar issues and a similar timeline may communicate regularly, perhaps with the support of an experienced facilitator and jointly problem-solve.

Many of the existing learning initiatives for health markets are global in nature and broadly encompass multiple market-based health models (e.g. the Center for Health Market Innovations, and the Private Sector in Health Thematic Working Group), however as communities tend to work better where there is extended exchange and significant trust between participants, it may be worthwhile to develop more geographically and/or thematically focused communities of practice, so as to enable more frequent and sustained interactions and hopefully faster and more effective learning cycles.

#### Institutional mechanisms to support learning

In some sectors there has been a strong history of self-organization for learning. For example, the micro-credit field has been very effective at building institutions to support communities of practice through organizations such as CGAP, The Microfinance Information Exchange and Imp-Act. To-date the institutions to support communities of practice around health markets have largely been dominated by global (northern) actors, and have centered on “experts” rather than practitioners. However communities of practice are most likely successful when they remain focused on practitioner experiences and needs [4] and thus require leadership from within the community, albeit from expert community members.

An alternative approach is that of an innovation broker that has been defined as “an organization acting as a member of a network of actors […] that is focused neither on the organization nor the implementation of innovations, but on enabling other organizations to innovate” [41]. Such brokers can help identify needs, mutual interests and connect different actors to each other. In the context of health markets, an innovation broker could help to identify learning needs and mutual interests across different market actors, and coordinate learning events. Local organizations, with extensive experience in health markets would be best placed to play this role. Access Health in India and the Philippine Institute for Development Studies, with support from CHMI, are beginning to take up this challenge – giving awards for outstanding innovators in health markets, and fostering linkages between innovators, policymakers, and funders. It is critical that social objectives are strongly reflected in their mission, and that part of their mandate is to set up learning systems so that positive adaptations get pursued, rather than simply those that serve the powerful.

A further alternative organizational form for the consolidation and dissemination of tacit knowledge may be through hybrid organizations that combine detailed market knowledge with a mission to support good practice. The US-based Advisory Board Company (a membership based private company, with a strong social mandate to improve hospital performance) could be an interesting model to explore in this respect. Such approaches may hold the promise of developing more timely evidence that is better geared to the needs of implementers than much traditional academic research.

## Conclusions

Enhancing learning about the characteristics of health markets, and how interventions such as social franchising or regulation affect their operations, is key to better decision-making. Policy-makers, social entrepreneurs and the corporate sector all need better evidence regarding health markets. This paper has identified three broad areas where targeted investments could move the health market learning agenda forward.

First, a concerted initiative is needed to strengthen data platforms for health markets. Currently data regarding health markets is fragmented and rarely brought together in a comprehensive fashion. Using specific countries as pilots, existing information sources could be reviewed, relevant variables identified and collated in a health market data platform. Such a data platform could combine information from routine information systems, household surveys, facility surveys, expenditure tracking surveys, market research and demographic and health surveillance sites. Concurrent with this collation of existing information sources, new initiatives should be piloted that strive to strengthen market data availability: for example initiatives could seek to improve private provider participation in health information systems, or experiment with new ways to capture data on specific geographic health markets within a country.

Policies promoting universal health coverage constitute a significant opportunity to build understanding of health markets. There is a need to ensure that appropriate data collection systems are embedded within payor management information systems (for example the data collected through routine claims, accreditation, provider empanelment processes etc.) as such systems are established in low and middle income countries.

Second, new approaches and increased investment in rigorous evaluations of health market interventions are critical. Evaluations need to better reflect and have sharper tools for analyzing market contexts, including the broader market impact of specific interventions. They also need to be able to capture the evolution of market interventions over time, and enable more real-time learning from such interventions. While evaluation designs for specific business models (such as social franchising arrangements) are relatively well established, appropriate approaches to evaluating policies or regulations intended to shape the market environment and change organizational incentives require further development. According to good evaluation practice, evaluators should be somewhat separate from implementers, but there is also a need for strong communication between the two so that evaluation designs are responsive to the ongoing evidence needs of implementers, and so opportunities for important empirical research can be identified in advance to enable sound methodology. If greater investment in evaluation is to be made, then it may make sense to identify evaluation priorities and target investments to address specific questions.

Finally, communities of practice hold much promise in terms of helping practitioners to address challenges in policy implementation or business innovation. More geographically and thematically focused communities of practice are needed, especially ones that encompass diverse stakeholders. Regional “innovation brokers” may be able to facilitate and support the development of such communities of practice. Given how nascent our understanding of communities of practice is, it will be important to evaluate what works and what does not work with respect to such communities.

While this paper has focused on the challenges to learning in health markets, we are optimistic that investment to address these challenges and accelerate learning can ultimately bring about significant improvements in the functioning of health markets, especially for the poor.

## Endnotes

^a^Notable examples include the Center for Health Market Innovation, HANSHEP, and the Private Sector Working Group of the SHOPS project. Other initiatives (such as HealthUnbound, the International Partnership for Innovative Health Care Delivery, Harmonization for Health In Africa and the UN Secretary General’s Innovation Working Group for Every Woman, Every Child) have interests that overlap with health markets, even if it is not their core focus.

^b^There are some ongoing studies of this nature for example BU faculty (with support from CHMI) are examining the impact on the market of the entry of the MedPlus Retail Pharmacy chain in Andrha Pradesh and what happens to price and quality in the non-chain pharmacies as a result of the new competition. In Bangladesh ICDDRB is working through the Future Health Systems Project to examine market dynamics around the introduction of a new m-health scheme.

^c^See for example the Health and Wellness Reports of Euromonitor.

^d^SECI refers to the acronym for the different stages in the process of knowledge acquisition and formalization, namely: Socialization (where tacit knowledge is shared); Externalization (where tacit knowledge is converted into explicit knowledge); Combination (where different forms of formal knowledge come together) and Internalization (where individuals reflect and absorb the new developed knowledge).

## Abbreviations

CGAP: The Consultative Group to Assist the Poor; CHMI: The Center for Health Market Innovations; GNP: Gross National Product; LMIC: Low and Middle Income Countries; NGO: Non-governmental organization; NHA: National Health Accounts; PETS: Public Expenditure Tracking Survey; SECI: Socialization, Externalization, Combination and Internalization; TB: Tuberculosis.

## Competing interests

The authors declare that they have no competing interests.

## Authors’ contributions

SB drafted the first version of the manuscript. GL, JK and HL all critically reviewed the manuscript and contributed additional text on relevant points. All authors reviewed and approved the final version of the manuscript.
